# Genetic diversity in two leading *Plasmodium vivax* malaria vaccine candidates AMA1 and MSP1_19_ at three sites in India

**DOI:** 10.1371/journal.pntd.0009652

**Published:** 2021-08-09

**Authors:** Sonal Kale, Veena Pande, Om P. Singh, Jane M. Carlton, Prashant K. Mallick

**Affiliations:** 1 Parasite-Host Biology Group, National Institute of Malaria Research, Indian Council of Medical Research, New Delhi, India; 2 Department of Biotechnology, Kumaun University, Nainital, India; 3 Center for Genomics and Systems Biology, Department of Biology, New York University, New York city, New York, United States of America; 4 Department of Epidemiology, School of Global Public Health, New York University, New York city, New York, United States of America; University of Liverpool, UNITED KINGDOM

## Abstract

*Plasmodium vivax*, a major contributor to the malaria burden in India, has the broadest geographic distribution and shows higher genetic diversity than *P*. *falciparum*. Here, we investigated the genetic diversity of two leading *P*. *vivax* vaccine candidate antigens, at three geographically diverse malaria-endemic regions in India. *Pvama1* and *Pvmsp1*_*19*_ partial coding sequences were generated from one hundred *P*. *vivax* isolates in India (Chennai n = 28, Nadiad n = 50 and Rourkela n = 22) and ~1100 published sequences from Asia, South America, North America, and Oceania regions included. These data were used to assess the genetic diversity and potential for vaccine candidacy of both antigens on a global scale. A total of 44 single nucleotide polymorphism (SNPs) were identified among 100 Indian *Pvama1* sequences, including 10 synonymous and 34 nonsynonymous mutations. Nucleotide diversity was higher in Rourkela and Nadiad as compared to Chennai. Nucleotide diversity measures showed a strong balancing selection in Indian and global population for domain I of *Pvama1*, which suggests that it is a dominant target of the protective immune response. In contrast, the *Pvmsp1*_*19*_ region showed highly conserved sequences in India and across the Oceania, South America, North America and Asia, demonstrating low genetic diversity in the global population when compared to *Pvama1*. Results suggest the possibility of including *Pvmsp1*_*19*_ in a multivalent vaccine formulation against *P*. *vivax* infections. However, the high genetic diversity seen in *Pvama1* would be more challenging for vaccine development.

## Author summary

*Plasmodium vivax* is the dominant malaria species in several countries, and a safe and effective vaccine against the species is needed if progress is to be made towards malaria control and elimination. The *P*. *vivax* Apical Membrane Antigen 1 (AMA1) ectodomain and the C-terminal region (19 kDa) of the Merozoite Surface Protein 1 (MSP1) are important malaria vaccine antigens. However, antigenic diversity remains a critical factor for acquisition of immunity and also a major barrier for development of an effective vaccine. Here, investigations on the genetic diversity of two blood stage vaccine candidate antigens, Merozoite surface protein 1_19_ and Apical membrane antigen 1, of *P*. *vivax* from three malaria endemic regions of the India was attempted and compared with global population. High genetic variation was observed in *Pvama1* domain I, which has been reported as a dominant target of host immune response. In contrast, *Pvmsp1*_*19*_ showed highly conserved sequences from three geographically diverse populations of India and globally, which further suggests *Pvmsp1*_*19*_ as an extremely conserved protein. Collectively, the diversity data generated in this study provides a baseline data towards development of a broadly efficacious *Pvama1* and *Pvmsp1*_*19*_ based vaccine.

## Introduction

*Plasmodium vivax* is a major malaria species which affects a large part of the world’s population [1]. Worldwide, more than 2.5 billion people live in high *P*. *vivax* malaria transmission areas and its burden has been increased as compared to *P*. *falciparum* over the last few years [1]. India reported largest drop in malaria cases in 2019, however in the WHO South-East Asia Region about 86% of malaria deaths were accounted from India [2]. *P*. *vivax* has a higher genetic diversity than the most common species *Plasmodium falciparum* indicating better fitness and potential to escape the immune response [3]. In addition, *P*. *vivax* resistance to the antimalarial drug chloroquine in various countries including India has raised a threat on malaria elimination [4]. India’s diverse geography and climate are ideal for the transmission of multiple *Plasmodium* species and have augmented the malaria burden in India [5]. The two-malaria species *P*. *vivax* and *P*. *falciparum* are unevenly distributed but also co-circulate in several Indian states. In the southern states (e.g. Tamil Nadu), *P*. *vivax* is the dominant malaria parasite species [6]. Eastern states like Odisha reported 47% of all *P*. *falciparum* cases and state like Gujarat (from the western part of India) showed the prevalence of both species, including mixed species infections [6]. To control malaria in such diverse regions, new tools including safe and effective vaccines are needed for malaria elimination, although extreme diversity in leading vaccine candidate antigens remains a major barrier for their development.

Hundreds of malaria vaccine candidates have been characterized, of which Apical membrane antigen 1(AMA1) and Merozoite surface protein 1(MSP1) are the most promising blood-stage vaccine candidates for *P*. *falciparum* and *P*. *vivax* [7, 8]. *P*. *vivax* Apical membrane antigen 1 (*Pvama1*), required for both merozoite invasion of erythrocytes and sporozoite invasion of hepatocytes [9], is an antigen of 66 kDa, secreted by microneme organelles and transported to the parasite’s surface just prior to erythrocyte invasion. The 1686 bp coding region of *Pvama1* codes for a cysteine-rich ectodomain, a conserved cytoplasmic region, and a transmembrane region [10]. The *Pv*AMA1 ectodomain is divided into three subdomains by disulfide bridges referred to as Domain I (DI), Domain II (DII) and Domain III (DIII) [11]. The ectodomain of *Pv*AMA1 is highly immunogenic and high antibody titers against the ectodomain have been reported in natural infections, which effectively inhibit erythrocyte invasion [9, 10]. DI and DII have been reported to be highly polymorphic and serological studies showed DII as most immunogenic [12].

*Plasmodium vivax* merozoite surface protein 1 (*Pv*MSP1) is a highly abundant protein present on the surface of merozoites. Initially it is synthesized as a high molecular weight precursor, which is further processed to various low molecular weight fragments [13]. The C terminal MSP-1_19_ fragment contains two epidermal growth factor-like domains that have been shown to be efficient blood-stage vaccine candidates [14]. At the time of red blood cell invasion, only MSP-1_19_ remains on the surface of the merozoite and participates in the initial adhesion to the reticulocyte [15]. Several studies have reported that acquired antibody against MSP-1_19_ can inhibit parasite growth [16–19] and are associated with protective immunity against infection [20–23].

To develop an effective malaria vaccine which can work in diverse regions of the world, it is important to study genetic diversity of vaccine candidates from geographically diverse regions to include alleles that can induce the immune response and cover the antigenic diversity of *P*. *vivax* population [24]. India contributes significantly in *P*. *vivax* malaria burden from Southeast Asia region, however, genetic diversity in *Pv*AMA1 and *Pv*MSP1_19_ vaccine candidate antigens from malaria endemic region of India has rarely been done [25–27]. Therefore, aim of this study were to investigate the genetic diversity of two leading vaccine candidate antigens *Pv*AMA1 and *Pv*MSP1_19_ kDa region from three geographically diverse malaria endemic regions of India. Published *Pvama1* and *Pvmsp1*_*19*_ sequences from the global parasite population and sequences generated in this study was analyzed to identify genetic polymorphism and selective forces acting on these genes_._ The results of present study thus have important implications in *Pvama1* and *Pvmsp1*_*19*_ based vaccine designing.

## Methods

### Ethics statement

The clinical samples collected in this study received ethical approval from the Institutional ethic committee of ICMR-NIMR in New Delhi, India and Institutional Review Board at New York University, School of Medicine in New York, New York, USA. Samples were collected after written consent was obtained from all participants.

### Study sites and sample collection

Samples were collected as part of a U.S. National Institutes of Health Center for the Study of Complex Malaria in India (CSCMi) project [5] from three geographically diverse malaria-endemic regions of India namely Chennai, Nadiad, and Rourkela (S1 Fig). The detailed description of the study sites has been given previously [28]. Briefly, Chennai is the largest city of the southern state of Tamil Nadu, with *P*. *vivax* as the dominant species during the malaria season in July and October. Samples were collected at Besant Nagar Malaria Clinic, and in cross-sectional surveys conducted in the coastal fishing community, including a few slums and middle and upper-class urban dwellings. Nadiad is a small town located in the central part of Gujarat state, which is considered as hypo-endemic for *P*. *vivax* and *P*. *falciparum*, with a slightly higher prevalence of *P*. *vivax*. Samples were collected at the Malaria Clinic, located in the Civil Hospital of Nadiad and in cross-sectional surveys conducted in the rural area of Nadiad town. The city of Rourkela located in the Sundargarh district of the eastern state of Odisha, where *P*. *falciparum* is the major infecting malaria species, displays meso to hyper endemic transmission in this region. Samples were collected at a malaria clinic in a suburb of Rourkela and cross-sectional surveys were conducted in a forest area of Sundargarh.

*P*. *vivax* infection was determined by RDT and microscopic examination of thick and thin blood smear. From each individual 2–3 ml of blood was collected in EDTA vacutainers (Thermo Fisher, Massachusetts, USA). Samples were centrifuged at room temperature at 1500 g for 15 min. Plasma and platelets were removed and stored at -80°C. DNA was extracted from infected red blood cells using DNA Blood MIDI kit (Qiagen, California, USA). A total of 100 *P*. *vivax* samples collected from infected individuals in Chennai (C, n = 28), Nadiad (N, n = 50) and Rourkela (R, n = 22) between January 2013 and May 2015 were used in this study (S1 Fig). *P*. *vivax* infections were confirmed by species-specific nested-polymerase chain reaction (nPCR) at each study site [6], and reconfirmed by the same nPCR at National Institute of Malaria Research, New Delhi.

### PCR amplification and sequencing

Primers were designed for *Pvama1* (PVX_092275) and *Pvmsp1*_*19*_ (PVX_099980) with Primer3 (version0.4.0) using the *P*. *vivax* Sal-1 genome reference from the malaria database PlasmoDB (www.PlasmoDB.org). Two primer sets were designed for *Pvama1* and one primer set was designed for C-terminal, 19kDa region of the *Pvmsp1*gene (S1 Table) and synthesized by Integrated DNA Technologies (IDT), India.

**Fig 1:**
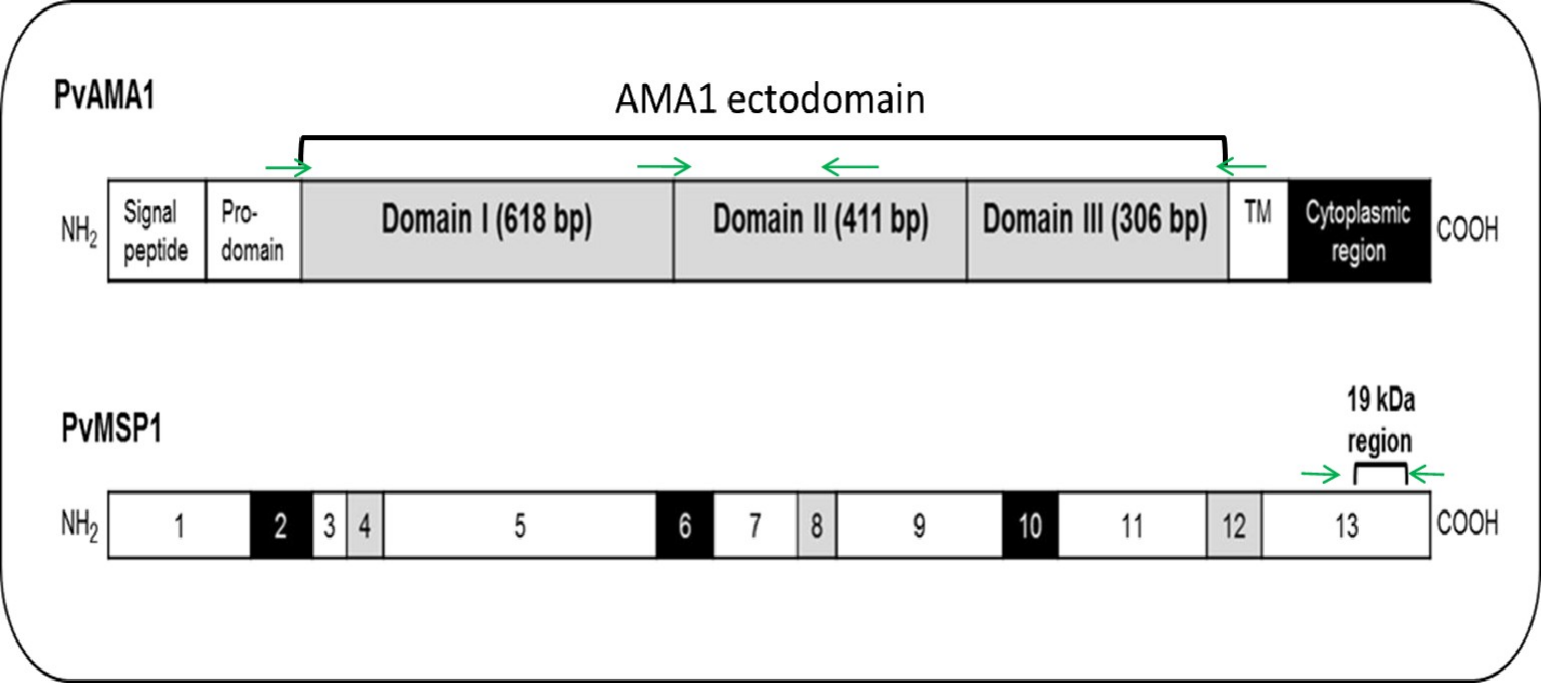
*Pvama1* and *Pvmsp1*_*19*_ region targeted for sequencing. Schematic diagram of the structure of *Pvama1* and the *Pvmsp1*_*19*_ region studied. Primer location is indicated with green arrows.

The *Pvama1* complete ectodomain (corresponding to nucleotide 127–1461 encoding amino acid 43–487 relative to Sal-I, PVX_092275) was amplified using two sets of primers (Fig 1). The amplifications were performed in a 25μl volume reaction with 1.5μl of DNA isolated from clinical isolates as the template with GoTaq Green PCR Master Mix (Promega), and 0.2 μM forward and reverse primers. The reaction was run at 94^o^c for 5 min, followed by 35 cycles of 94^o^c for 30 s, 55^o^c for 1 min, and 62^o^c for 3 min, with a final extension at 62^o^c for 10 min. PCR fragments were visualized by electrophoresis on 1.5% agarose gel, using a 1-kb DNA ladder (Promega).MSP-1_19_C terminal region(corresponding to nucleotide 4921–5187 encoding amino acid 1641–1729 relative to Sal-I PVX_099980) was PCR amplified (Fig 1) as above and the thermo profile was 94^o^c for 5 min, followed by 35 cycles of 94^o^c for 30 s, 60^o^c for 1 min, and 62^o^c for 3 min, with a final extension at 62^o^c for 10 min. PCR fragments were visualized by electrophoresis on a 2% agarose gel, using a 100-kb DNA ladder (Promega). PCR products were purified using the Exonuclease-I and Shrimp Alkaline Phosphatase (Exo-SAP, Thermo Fisher Scientific) followed by sequencing reactions in both forward and reverse directions (2x coverage) by a commercial sequencing facility using ABI BigDye Terminator Cycle Sequencing kit on an ABI 3730XL automatic DNA Analyzer (Macrogen, Seoul, Korea).

### DNA sequence assembly and polymorphism analysis

Raw sequence data were assembled and edited using the SeqMan (DNASTAR, Lasergene, SeqMan Pro). The DNA sequence chromatograms were carefully inspected to identify the SNP’s. All *Pvama1* sequences (n = 100) and *Pvmsp1*_*19*_ sequences (n = 100) were aligned to the *P*. *vivax* Sal-I reference strain sequence. A 1335 bp region encompasses the complete ectodomain (DI, DII & DIII) of *Pvama1*, and 266 bp region of *Pvmsp1*_*19*_ was assembled excluding primer regions.

To investigate the global genetic diversity of *Pvama1* and *Pvmsp1*_*19*_, published sequences were retrieved from GenBank (S2 Table). A total of 616 *Pvama1* complete gene sequences from eight distinct subpopulations, and sequences of six primate-adapted *P*. *vivax* isolates used for vaccine research (Belem, India VII, Palo Alto, North Korea, Indonesia XIX, Chesson) were retrieved from GenBank and added into the analysis. A total of 582 *Pvmsp1*_*19*_ sequences (full-length *Pvmsp1*_*19*_ gene sequence and *Pvmsp1*_*42*_ region sequence) from 14 distinct *P*. *vivax* subpopulations and two reference strains (Sal-I and Belem) were retrieved for the analysis. Combined, these represent *Pvama1* and *Pvmsp1*_*19*_ sequences from seven global regions: East Asia (South Korea, China and Myanmar border), South Asia (India, Sri Lanka, and Bangladesh), Southeast Asia (Thailand, Myanmar, Singapore, and Cambodia), West Asia (Iran, Turkey), South America (Venezuela, Brazil), North America (Mexico), and Oceania (PNG. Vanuatu).

All sequences were aligned by the CLUSTAL W program in MEGA6.0 [29] and nucleotide diversity analysis was performed in the DnaSP v5.10.01 software [30]. Several diversity statistics were calculated for each studied Indian population and the global data set of *Pvama1* and *Pvmsp1*_*19*_, which includes total number of polymorphic sites (S), nucleotide diversity (π), the number of haplotypes (h), haplotype diversity (Hd), the number of synonymous mutation (SP), non-synonymous mutation (NS), using a 100 bp sliding window with a 25 bp step size in DnaSP.

To test the neutral theory of evolution in *Pvama1* and *Pvmsp1*_*19*_, Tajima’s D, Fu and Li’s D and F were calculated in DnaSP. Further, to evaluate natural selection the McDonald-Kreitman (MK) test was performed [31].The sequence of *Plasmodium cynomolgi* AMA1 was used as the interspecies outgroup (GenBank accession no. XM_004222507.1) [32]. Wright’s fixation index *F*_ST_ by DnaSP was calculated to determine the degree of genetic variance in *Pvama1* and *Pvmsp1*_*19*_ kDa region among global isolates. The minimum number of recombination events (Rm), and linkage disequilibrium (LD) was calculated. The relationship between LD and nucleotide sites distance was plotted by using indices D’ and R^2^. To investigate the genetic relatedness among Indian and global *Pvama1* haplotypes, network analysis was performed using a haplotype frequency > 1. For *Pvmsp1*_*19*_ all Indian and global haplotypes were used and analysis were performed using Network software version 5.0.0.3 [33]. Mutation sites in *Pv*AMA1 ectodomain were visualized as a three dimensional structure of *Pv*AMA1 (PDB ID: 1W81) by Pymol [34].

### Accession numbers

Sequences generated in this study have been deposited in GenBank under the accession numbers: 100 *Pvama1* sequences MH657021—MH657120, and 100 *Pvmsp1*_*19*_ sequences MH657121—MH657220

## Results

### Genetic diversity and signatures of selection within *Pvama1* at three sites in India

A 1,335 bp region of *Pvama1* was amplified from 100 *P*. *vivax* isolates collected from Chennai (C; n = 28), Nadiad (N; n = 50) and Rourkela (R; n = 22). The average number of nucleotide differences (K) were higher in Rourkela and Nadiad population when compared to Chennai (Table 1). A total of 44 SNP’s was identified among these 100 Indian *Pvama1* sequences, including 10 synonymous and 34 nonsynonymous mutations. Nucleotide diversity was higher in Rourkela and Nadiad population and lower in Chennai Population (Table 1).

**Table 1:** Estimates of genetic diversity and test of neutrality for *Pvama1* in three Indian populations.

PvAMA1	Region	N	S	M	K	π ± SD	NS	SP	H	Hd ± SD	Tajima’s D	D	F	MK
**Domain I**	C	28	23	24	5.698	0.009 ± 0.002	18	6	11	0.741 ± 0.084	-0.273	0.624	0.399	0.000252***
	N	50	24	26	9.413	0.015 ± 0.000	17	6	31	0.971 ± 0.011	2.041*	1.78**	2.226**	0.000477***
	R	22	24	26	9.407	0.015 ± 0.001	19	7	18	0.978 ± 0.021	1.212	0.985	1.230	0.000353***
	Total	100	26	28	8.905	0.014 + 0.000	19	6	45	0.938 ± 0.016	1.952*	1.211	1.793*	0.000136***
**Domain II**	C	28	13	14	3.156	0.008 ± 0.000	11	3	10	0.844 ± 0.044	-0.415	-1.329	-1.226	0.001561**
	N	50	15	16	2.564	0.006 ± 0.000	13	3	19	0.896 ± 0.03	-0.873	-1.032	-1.158	0.000662***
	R	22	10	11	2.844	0.007 ± 0.000	9	2	12	0.939 ± 0.028	-0.199	0.528	0.366	0.003571**
	Total	100	15	16	2.83	0.007 ± 0.000	13	3	24	0.900 ± 0.017	-0.235	0.582	0.336	0.00024***
**Domain III**	C	28	1	1	0.071	0.000 ± 0.000	1	0	2	0.071 ± 0.065	-1.151	-1.659	-1.747	0.472222
	N	50	3	3	0.599	0.002 ± 0.000	2	1	4	0.543 ± 0.045	-0.218	-0.413	-0.412	0.592857
	R	22	3	3	0.666	0.002 ± 0.000	2	1	4	0.571 ± 0.081	-0.491	-1.309	-1.247	0.592857
	Total	100	3	3	0.506	0.002 + 0.000	2	1	4	0.460 ± 0.045	-0.228	0.827	0.579	0.592857
**Ectodomain**	C	28	37	39	8.926	0.007+ 0.000	30	9	13	0.868 ± 0.048	-0.407	-0.273	-0.372	0.000002***
	N	50	42	45	12.576	0.009 + 0.000	32	10	36	0.983 ± 0.009	0.863	0.619	0.843	0.000002***
	R	22	37	40	12.918	0.010 + 0.000	30	10	20	0.991 ± 0.017	0.692	0.671	0.792	0.000004***
	Total	100	44	47	12.241	0.009 +0.000	34	10	55	0.968 ± 0.009	1.102	1.211	1.400	0.000001***

**Abbreviation**: N numbers of samples, S number of polymorphic sites, M the total number of mutations, K the average number of nucleotide differences, π pairwise nucleotide diversity, SD standard deviation, NS number of nonsynonymous mutation, SP number of synonymous mutation, H number of haplotypes, HD haplotype diversity, Tajima’s D test, D Fu and Li’s D value, F Fu and Li’s F value, MK McDonald-Kreitman test

* P<0.05

** 0.001<P<0.01

*** P<0.001

Natural selection was assessed across the region encoding the *Pv*AMA1 ectodomain for Indian population. Balancing selection was observed in Nadiad and Rourkela population, whereas Chennai population showed no evidence of balancing selection. The positive value of Tajima’s D, Fu and Li (F and D) for Nadiad and Rourkela populations indicates deviation from neutral evolution and the tendency for diversifying selection in the corresponding regions. The negative Tajima’s value, Fu and Li (F and D) value for Chennai population indicates purifying selection. McDonald-Kreitman test showed that the entire *Pvama1* ectodomain, particularly DI, had more nonsynonymous substitution as compared to synonymous substitution.

The DNA sequence polymorphism analysis were performed for each domain of *Pvama1*. The average number of nucleotide differences (K) for the domain I was higher in the Nadiad and Rourkela population and lower in the Chennai population (Table 1). A total of 45 haplotypes (C = 11, N = 31, R = 18) were identified among Indian isolates in domain I, determined an extremely high level of haplotype diversity (Hd = 0.938). Haplotype diversity was higher in Rourkela and Nadiad in comparison to Chennai population. The significant positive value of Tajima’s D for Nadiad population, indicates deviation from neutral evolution and the tendency for diversifying selection, however, these values were not statistically significant for Rourkela and Chennai population. The DNA sequence polymorphism analyses of domain II indicated that the average number of nucleotide differences (K) for domain II was highest in the Chennai population and lowest in Nadiad and Rourkela populations. A total of 24 haplotypes (C = 10, N = 19, R = 12) were identified among Indian isolates in domain II and high level of haplotype diversity (Hd = 0.900) was observed. Haplotype diversity was highest in the populations from Rourkela and Nadiad and lowest in isolates from Chennai. For domain III the average number of nucleotide differences (K) was higher in the Rourkela and Nadiad populations and lowest in isolates from Chennai (Table 1). A total of four haplotypes (C = 2, N = 4, R = 4) were identified among these isolates, which demonstrated a low level of haplotype diversity (Hd = 0.46) (Table 1). Haplotype diversity was higher in the Rourkela and Nadiad populations and lowest in the Chennai population.

### Polymorphic amino acids residues within *Pv*AMA-1 at three sites in India

**Fig 2:**
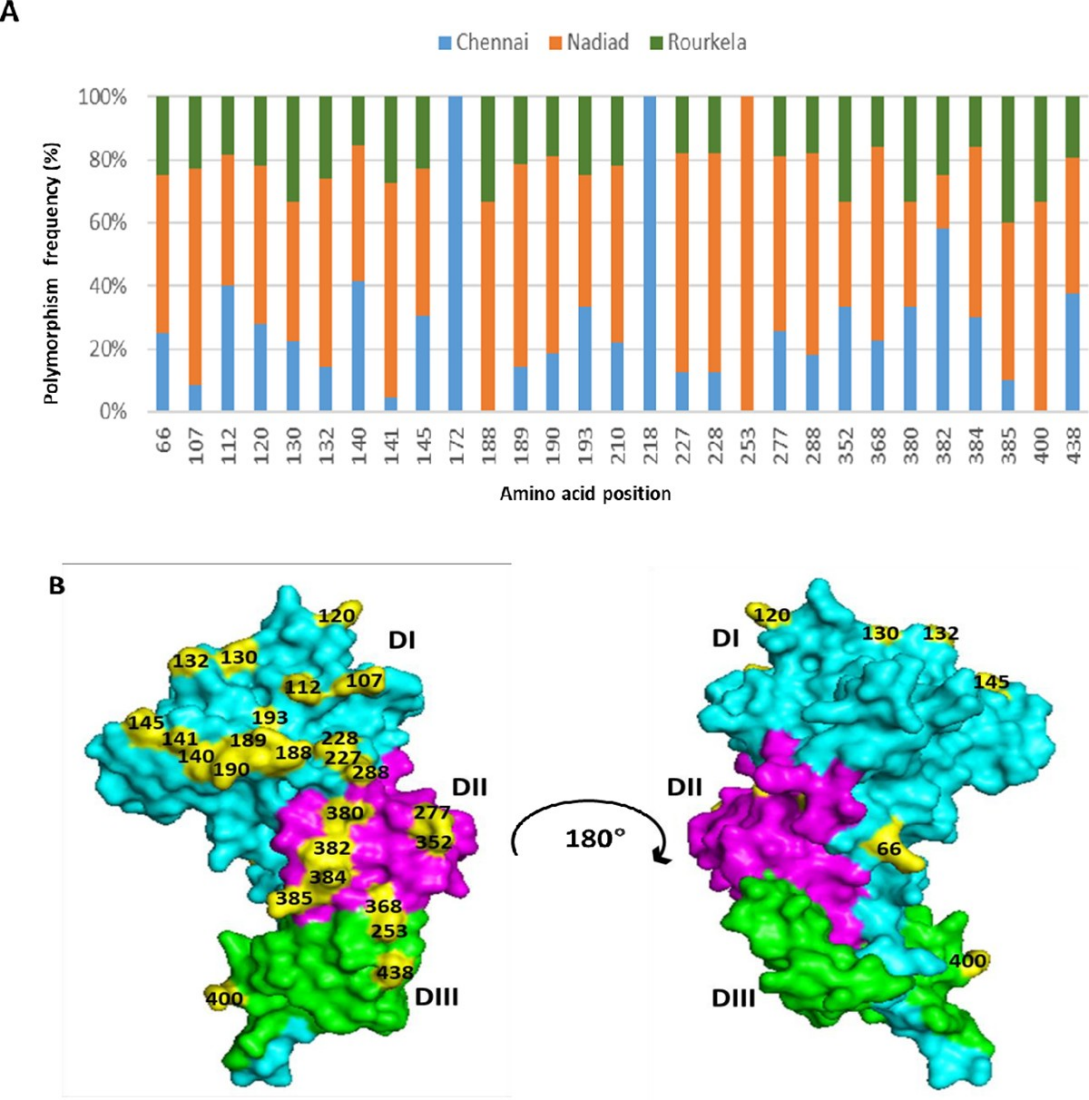
Frequency and location of polymorphic sites found in *Pv*AMA1 from Indian isolates. **(A)** The frequency of amino acid polymorphism at study sites Chennai, Nadiad and Rourkela are represented in blue, orange and green respectively. (**B)** Based on the *Pv*AMA1 crystal structure (PDB ID: 1W81) Domain I, II and III are colored in cyan, magenta and green respectively. Polymorphic amino acid residues at each domain are numbered and colored in yellow.

The frequency of amino acid polymorphisms at each study site and for each domain were calculated (Fig 2A). A total of 29 amino acid changes were detected in the *Pv*AMA1 ectodomain. A total of 18 amino acid changes were found in Domain I, nine in Domain II and two amino acid changes were found in Domain III of *Pv*AMA1. DI was found to be the most variable as compared to DII and DIII. A three-dimensional model of *Pv*AMA1 was generated to understand the location of the polymorphic residues on the *Pv*AMA1 active face and silent face, represented in Fig 2B. Only one polymorphic residue on position 66 was located on the opposing face of the *Pv*AMA1 molecule. Other residues were located on the active face of the *Pv*AMA1 structure (Fig 2B).

### Global genetic diversity in *Pvama1* and evidence of selection

To study *Pvama1* global genetic diversity, sequence data obtained from Chennai, Nadiad, and Rourkela was then compared to published Indian sequences (n = 11) as well as seven geographically diverse *P*. *vivax* populations, including from Thailand (n = 231), Sri Lanka (n = 23), Iran (n = 29), Korea (n = 66), China-Myanmar border (n = 73), Venezuela (n = 73), PNG (n = 102), and a set of monkey adapted reference isolates (n = 8) (Table 2).

**Table 2:** Summary population genetic data for *Pvama1* and *Pvmsp1*_*19*_ region in worldwide populations.

Region	Country	n	S	M	k	NS	SP	Π± SD	H	Hd ± SD	Tajima’s D		D	F
**PvAMA-1**														
South Asia	India	111	43	45	12.269	36	9	0.009 ± 0.000	62	0.972 + 0.008	1.097	0.278		0.737
	Sri Lanka	23	34	32	10.13	27	5	0.007 ± 0.001	15	0.949 ± 0.028	0.262	0.845		0.779
East Asia	China-Myanmar	73	43	46	10.895	33	13	0.008 ± 0.000	41	0.958 ± 0.014	0.495	0.772		0.796
	Korea	66	57	57	7.68	35	22	0.005 ± 0.000	27	0.950 ± 0.013	-1.204	-0.835		-1.169
West Asia	Iran	29	40	43	12.936	34	9	0.009 ± 0.000	29	1.000 ± 0.009	0.675	1.160		1.182
Southeast Asia	Thailand	231	52	46	11.927	34	12	0.009 ± 0.000	94	0.934 ± 0.012	0.768	0.131		0.502
South America	Venezuela	73	28	26	9.679	20	6	0.007 ± 0.000	18	0.909 ± 0.000	1.960	0.949		1.585
Oceania	PNG	102	40	39	10.78	32	7	0.008 ± 0.000	85	0.996 ± 0.002	0.950	1.355		1.430
	Reference strains	8	45	47	16.107	37	10	0.012 ± 0.001	8	1.000 ± 0.003	-0.386	-0.67		-0.677
	Total	716	139	129	13.568	85	41	0.010 ± 0.000	352	0.989 ± 0.001	-0.87065	-5.363**		-3.514**
**PvMSP-119**														
South Asia	India	128	-	-	-	-	-	-	1	-	-	-		-
	Sri Lanka	106	3	3	0.317	1	2	0.001 + 0.000	5	0.228 + 0.054	-0.794	-0.599		-0.776
	Bangladesh	5	1	1	0.4	0	1	0.001 + 0.001	2	0.400 + 0.237	-0.816	-0.816		-0.771
East Asia	South Korea	22	1	1	0.091	1	0	0.0003 + 0.000	2	0.091 + 0.081	-1.162	-1.575		-1.678
	China	2	-	-	-	-	-	-	-	-	-	-		-
West Asia	Turkey	40	1	1	0.508	1	0	0.002 + 0.000	2	0.508 + 0.024	1.655	0.564		1.007
Southeast Asia	Thailand	209	2	2	0.199	1	1	0.001 + 0.000	3	0.198 + 0.034	-0.558	-1.233		-1.201
	Myanmar	29	2	2	0.202	1	1	0.000 + 0.000	3	0.197 + 0.095	-1.249	-0.726		-1.008
	Singapore	50	9	9	0.36	8	1	0.0014 + 0.001	7	0.228 + 0.079	-2.297***	-4.583**		-4.519**
	Cambodia	44	1	1	0.333	1	0	0.0012 + 0.000	2	0.333 + 0.073	0.623	0.555		0.663
South America	Brazil	8	-	-	-	-	-	-	-	-	-	-		-
North America	Mexico	35	-	-	-	-	-	-	-	-	-	-		-
Oceania	Vanuatu	2	-	-	-	-	-	-	-	-	-	-		-
	Reference Strains	2	-	-	-	-	-	-	-	-	-	-		-
	Total	682	14	14	0.228	10	4	0.00109 + 0.000	14	0.200 +0.020	-1.973*	-5.136**		-4.740**

**Abbreviation**: N numbers of samples, S number of polymorphic sites, M the total number of mutations, K the average number of nucleotide differences, π pairwise nucleotide diversity, SD standard deviation, NS number of nonsynonymous mutation, SP number of synonymous mutation, H number of haplotypes, HD haplotype diversity, Tajima’s D test, D Fu and Li’s D value, F Fu and Li’s F value, MK McDonald-Kreitman test

* P < 0.05

** P < 0.02

***P<0.01

**Fig 3:**
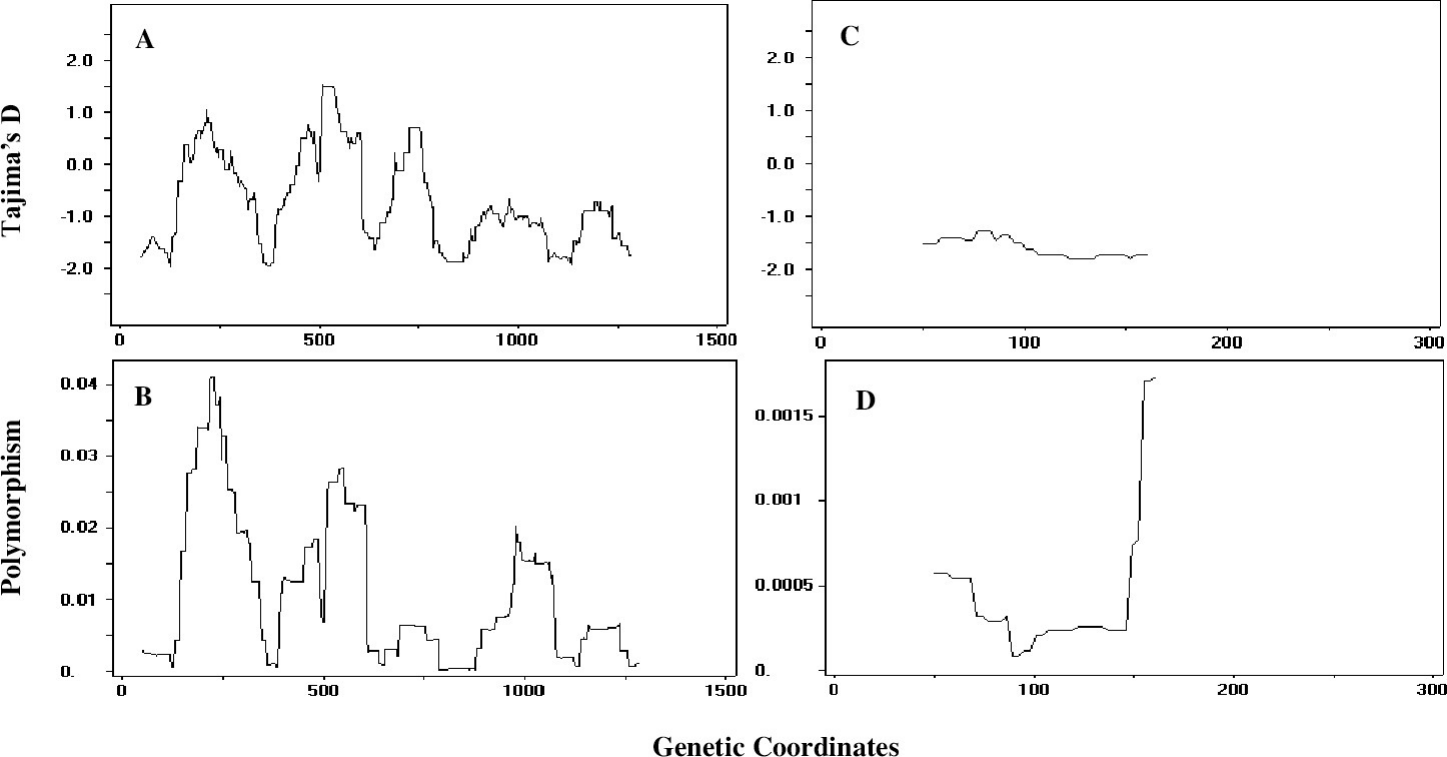
Tajima’s D and Nucleotide diversity across the *Pvama1* ectodomain and *Pvmsp1*_*19*_ kDa region in global isolates. Tajima’s D (A) and nucleotide diversity (B) were calculated across the *Pvama1* ectodomain for global isolates. A sliding window (100 bp window and 3 bp step size) was used to achieve a high-resolution analysis. *Pvmsp1*_*19*_ kDa region were analyzed for Tajima’s D (C) and nucleotide diversity (D) using a sliding window. All coordinates are based on Sal1 *Pvama1* and *Pvmsp1*_*19*_ reference sequences.

A total 716 *Pvama1* sequences here onwards referred as the “global” (including the 100 Indian sequences obtained in this study and 616 published sequences retrieved from GenBank described above) were used to determine nucleotide diversity and genetic differentiation. A total of 126 SNP’s was identified amongst the 716 global isolates, including 41 synonymous mutations and 85 nonsynonymous mutations. The average number of nucleotide difference (k) for the *Pvama1* ectodomain across global isolates was 13.568. The higher number of nucleotide differences was found in Iran and India and the least number of differences were seen in isolates from South Korea. The haplotype diversity for the entire *Pvama1* ectodomain of global isolates was (Hd = 0.9898). This value was higher in Iran, PNG, and India when compared to the other populations. The nucleotide diversity (π) was higher in Domain-I (DI) of the *Pvama1* among global isolates (Fig 3).

**Fig 4:**
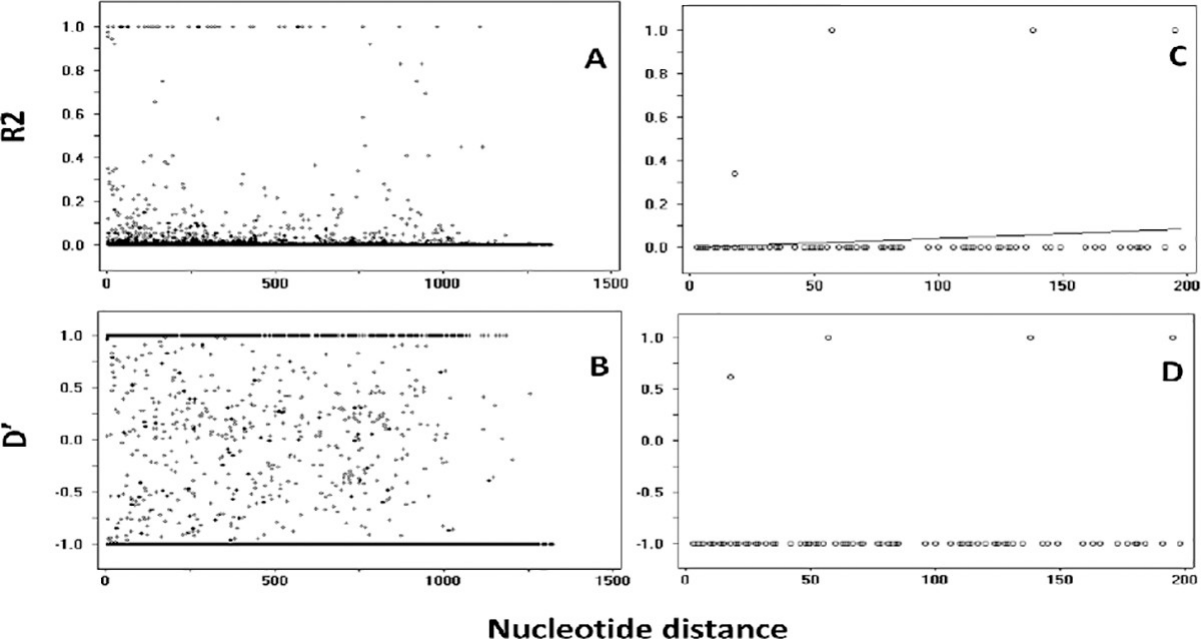
Linkage disequilibrium plots of R2 and D’ for *Pvama1* and *Pvmsp1*_*19*_ kDa region for global isolates. (A) Linkage disequilibrium plots of R2 and (B) D’ for *Pvama1* ectodomain, (C) linkage disequilibrium plots of R2 and (D) D’ for *Pvmsp1*_*19*_ kDa region were calculated by Fisher’s exact test.

We assessed selection in global population and the results of Tajima’s D, Fu and Li D and F statistics indicate balancing selection in *Pvama1* ectodomain in India, Sri Lanka, China Myanmar border, Thailand, PNG, Iran, Venezuela population. Only the South Korea population indicates negative Tajima’s D value which was not significant. For the global *Pvama1* isolates, the minimum number of recombination events, (Rm) estimated was 23 (Fig 4).

### Genetic diversity within *Pvmsp1*_*19*_ at three sites in India

Nucleotide sequence analysis of the 100 *Pvmsp1*_*19*_ (C = 28, N = 50, R = 22) sequences revealed that this region is highly conserved, and it didn’t show any polymorphic site in three geographically diverse malaria endemic regions of India.

### Global genetic diversity in *Pvmsp1*_*19*_ and evidence of selection

Although the *Pvmsp1*_*19*_ kDa region is a potential vaccine candidate, its global genetic diversity has not been carefully evaluated. To study the genetic diversity of *Pvmsp1*_*19*_, sequence data generated in the present study from India were compared to previously published sequences from India (n = 28), Thailand (n = 209), Sri Lanka (n = 106), Mexico (n = 35), Singapore (n = 50), Brazil (N = 11), Cambodia (n = 44), Myanmar (n = 28), Turkey (n = 40), South Korea (n = 22), China (n = 2), Bangladesh (n = 5), Vanuatu (n = 2), and two reference strains (Sal I and Belem).

A total 682 *Pvmsp1*_*19*_ sequences here onwards referred as “global” (including 100 Indian sequences obtained in this study and 582 published sequences retrieved from GenBank described above) were used to determine nucleotide diversity and genetic differentiation. There was no genetic or nucleotide diversity found in Indian, South American (Brazil), and Oceanian (Vanuatu) isolates.

**Fig 5:**
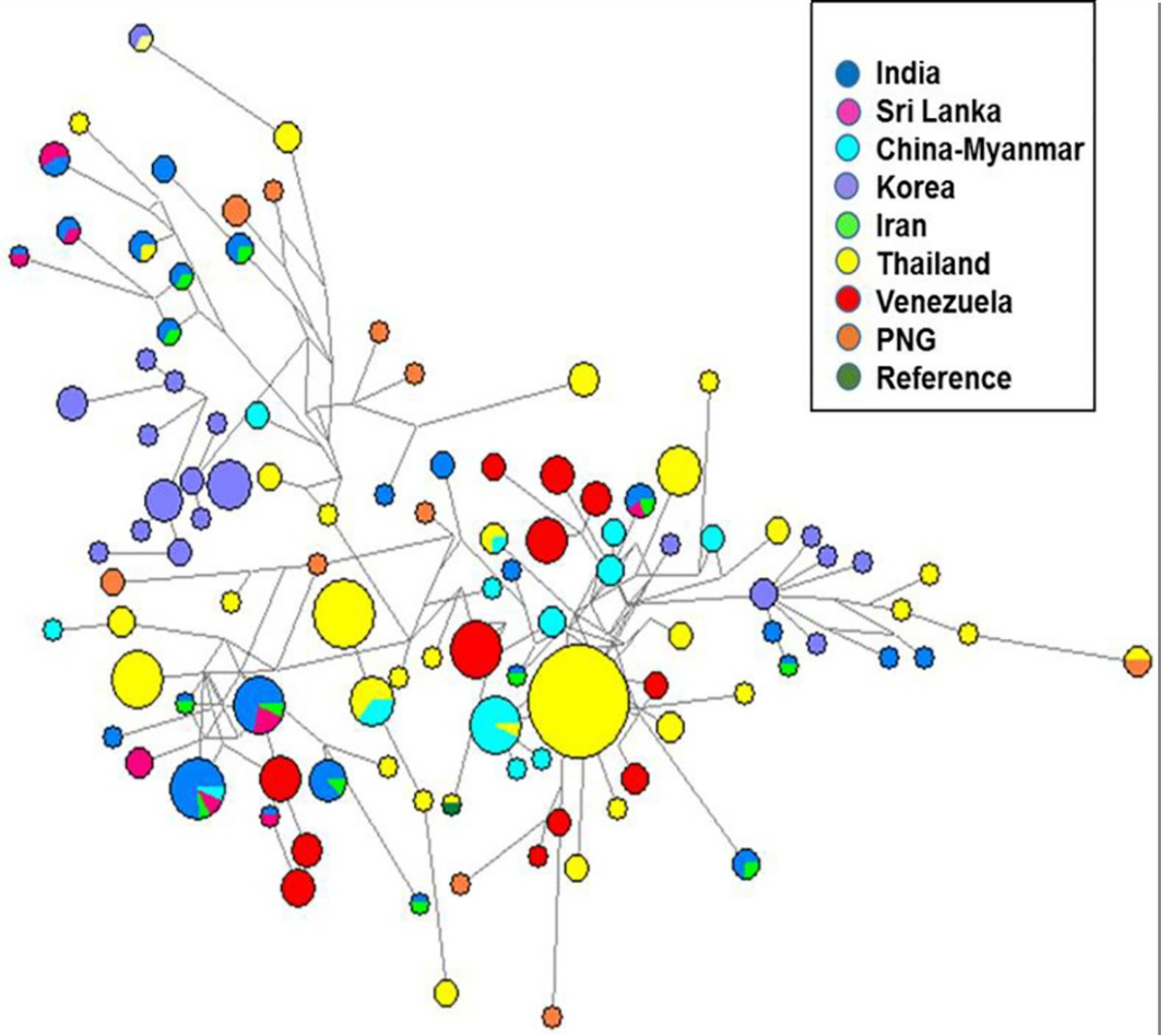
Median-joining network of *Pvama1* between eight geographically diverse populations. Haplotypes composed of nucleotide polymorphism in *Pvama1* ectodomain with a frequency > 1 were used to create a median-joining network. This network represents the mutational paths connecting *Pvama1* haplotypes that may explain the observed sequence diversity. Each node represents one haplotype, node size indicates haplotype frequency and node color corresponds to the country of origin. Line length is proportional to genetic distance.

A total of 14 SNP’s was identified among the 682 global sequences, including four synonymous mutations and ten nonsynonymous mutations. The average number of nucleotide difference (k) for the *Pvmsp1*_*19*_ kDa region across global isolates was 0.229. The highest number of nucleotide differences were found in Turkey (K = 0.508) and Bangladesh (0.4) while the least was seen in South Korea (K = 0.091) and Thailand (K = 0.181). The haplotype diversity (Hd) for the entire *Pvmsp1*_*19*_ region of global isolates was Hd = 0.2. This value was higher in Turkey (Hd = 0.508) than the other populations. The nucleotide diversity (π) of the entire *Pvmsp1*_*19*_ kDa region for global isolates was π = 0.00937 (Table 2). Overall nucleotide diversity observed across the global population indicated low nucleotide diversity in *Pvmsp1*_*19*_ region (Fig 5).

**Fig 6:**
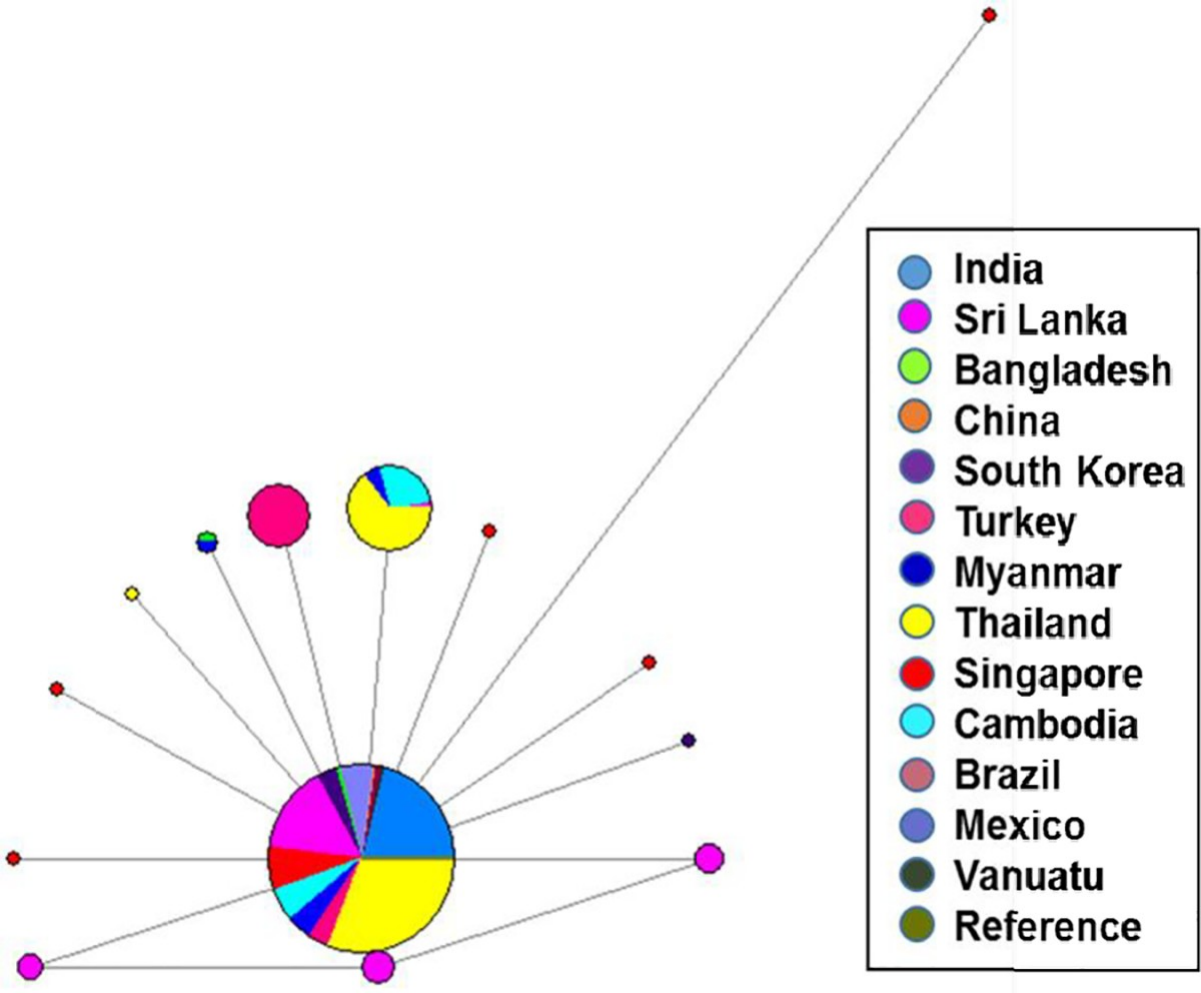
Median-joining network of *Pvmsp1*_*19*_ between thirteen geographically diverse populations. *Pvmsp1*_*19*_ sequences from diverse geographical regions were used to create a median-joining network. This network represents the mutational paths connecting *Pvmsp1*_*19*_ haplotypes that may explain the observed sequence diversity. Each node represents one haplotype, node size indicates haplotype frequency and node color corresponds to the country of origin. Line length is proportional to genetic distance.

The estimated Tajima’s D value was -1.97046 (P < 0.05), indicating that *Pvmsp1*_*19*_ is under purifying selection. The Fu and Li’s D and F values for *Pvmsp1*_*19*_ global isolates -5.13595 (P < 0.02) and -4.74044 (P < 0.02) were also negative. For the global *Pvmsp1*_*19*_ isolates, the minimum number of recombination events (Rm), estimated was 1. The LD index (R^2^) was also very low suggesting the absence of recombination events and low LD may have contributed to the low diversity in *Pvmsp1*_*19*_ (Fig 6).

Sequence analysis of the global data resulted in 14 different haplotypes with amino acid changes at 11 codons as aligned to Sal I. All 11 amino acid changes were dimorphic and region specific. Amino acid change 1706E was found only in Turkey (2010, 2012). We observed 8 amino acid changes specific to isolates from Singapore S1643P, D1649N, R1669G, C1681R, C1689R, A1697V, N1708D and P1721Q and one amino acid change specific to South Korea N1692K. Only one amino acid change (K1709E) was found in three different populations from Thailand, Cambodia and Myanmar.

### *F*_ST_ analysis

To understand population differences in *Pvama1* due to genetic variation among the three India populations, we calculated the *F*_ST_ value using *Pvama1* sequences from the Chennai, Nadiad and Rourkela. A moderate level of genetic differentiation (*F*_ST_ = 0. 143) was detected between Nadiad and Chennai isolates, a low level of genetic differentiation (*F*_ST_ = 0.075) was detected between the Rourkela and Chennai population, and the *F*_ST_ value observed between Nadiad and Rourkela (*F*_ST_ = -0.003) demonstrated a high degree of genetic similarity between these two populations.

*F*_ST_ values for the eight worldwide *Pvama1* populations with full-length ectodomain sequence were also calculated (Table 3). A high level of genetic differentiation was detected between the Indian and South Korean populations. A high *F*_ST_ value (0.36–0.54) was also detected for South Korea and other population. Moderate *F*_ST_ values (0.07–0.13) were detected among the India, China Myanmar, Thailand, Venezuela, PNG, and Sri Lanka populations. A negative *F*_ST_ value (-0.00687) was observed between Indian and Iranian isolates, showing a high degree of genetic similarity between these two populations.

**Table 3:** Geospatial differences in *Pvama1* and *Pvmsp1*_*19*_ sequences.

PvAMA-1	India	Sri Lanka	China Myanmar	South Korea	Iran	Thailand	Venezuela							
India														
Sri Lanka	0.07177													
China Myanmar	0.12941	0.23963												
South Korea	0.39192	0.54209	0.46673											
Iran	-0.00687	0.09753	0.10977	0.36529										
Thailand	0.12906	0.20857	0.03421	0.4687	0.11771									
Venezuela	0.13129	0.24976	0.12344	0.47253	0.09048	0.16639								
PNG	0.0958	0.22304	0.188	0.41695	0.10249	0.16961	0.23264							
**PvMSP-119**	**India**	**Sri Lanka**	**Bangladesh**	**China**	**South Korea**	**Turkey**	**Myanmar**	**Thailand**	**Singapore**	**Cambodia**	**Brazil**	**Mexico**
India												
Sri Lanka	0.06772											
Bangladesh	0	0.0311										
China	0	0.06772	0									
South Korea	0	0.05342	0	0								
Turkey	0.4359	0.33503	0.30178	0.4359	0.39591							
Myanmar	0.02469	0.04644	-0.03975	0.02469	0.01734	0.35682						
Thailand	0.09657	0.07249	0.03428	0.09657	0.06834	0.36922	-0.00873					
Singapore	0	0.03712	0	0	0	0.33246	0.0107	0.0425				
Cambodia	0.18605	0.12334	0.09407	0.18605	0.15222	0.35782	0.04064	0.0207	0.11045			
Brazil	0	0.06772	0	0	0	0.4359	0.02469	0.09657	0	0.18605		
Mexico	0	0.06772	0	0	0	0.4359	0.02469	0.09657	0	0.18605	0	
Vanuatu	0	0.06772	0	0	0	0.4359	0.02469	0.09657	0	0.18605	0	0

*F*_ST_ values of the thirteen worldwide *Pvmsp1*_*19*_ populations were evaluated (Table 3). *F*_ST_ values of India, Bangladesh, China, South Korea, Singapore, Brazil, Mexico, and Vanuatu approached zero, indicating a high degree of genetic similarity. Turkey showed a high *F*_ST_ value (0.33–0.43) when compared to India, China, Myanmar, Thailand, Singapore, Cambodia, Brazil, Mexico and Vanuatu populations, which represents a high degree of genetic distance. Moderate *F*_ST_ values (0.02–0.18) were detected when the Indian population was compared with population from Cambodia, Thailand, and Myanmar.

### Haplotype diversity

A total of 55 haplotypes were identified among Indian isolates of *Pvama1*, demonstrating an extremely high level of haplotype diversity across the three sites (Hd 0.968). The *Pvama1* data showed only 4 haplotypes (Hap1, Hap3, Hap5 and Hap7) shared among these three populations. One haplotype (Hap6) is shared between the Chennai and Rourkela populations, one haplotype (Hap8) is shared between the Chennai and Nadiad populations, and three haplotypes (Hap17, Hap16, and Hap34) are shared between the Nadiad and Rourkela isolates. We observed region specific haplotypes in Chennai (n = 6), Nadiad (n = 27) and Rourkela (n = 12) (S2 Fig).

Based on the polymorphism observed in the entire *Pvama1* ectodomain, a total of 352 haplotypes were identified in 716 global isolates with no matches to the Sal1 reference strain; only one isolate from Thailand matched to the Belem reference strain. A haplotype network formed using haplotypes with a frequency > 1 revealed a clearer network. Haplotype 2, haplotype 4 and haplotype 22 were shared among the Indian, Sri Lankan and Iranian populations with frequencies of 2.3, 1.9 and 0.69 respectively. Haplotypes 6, 7, 11 and 20 were shared among the Indian and Iranian population with a frequency of 1.1, 0.5, 0.5, and 0.2 respectively (Fig 5).

For the *Pvmsp1*_*19*_ kDa region, a total of 14 unique haplotypes were identified in 682 global isolates. Haplotype 1 matched to the Sal1 reference strain and was the predominant haplotype in the global population shared among India, Bangladesh, China, Brazil, Turkey, Thailand, South Korea, Sri Lanka, Singapore, Cambodia and Vanuatu, with a frequency of 89.2. Haplotype 2 was restricted to Turkey isolates with a frequency of 2.6. Haplotype 3 was shared among Thailand, Sri Lanka, Cambodia and Myanmar populations with a frequency of 4.9. Haplotype 4 was shared among Bangladesh and Myanmar with a frequency of 0.29, haplotype 5 was restricted to Thailand, haplotype 6–8 were restricted to Sri Lanka, haplotype 9–13 were restricted to Singapore and haplotype 14 was restricted to South Korea population with a frequency of 0.14–0.73 (Fig 6)

## Discussion

*Plasmodium vivax* remains a major cause of malaria in Southeast Asia region, particularly in India and thus must be targeted to achieve a malaria free India by 2030 [2, 35]. *P*. *vivax* is likely more difficult to eliminate than *P*. *falciparum* [36] due to its ability to relapse from long-lasting dormant liver stages, a shorter development cycle in mosquitoes, and a more genetically diverse genome [37–39]. Therefore, research towards malaria vaccine development targeting *P*. *vivax* must be prioritized. This is the first study from India that provides a comprehensive evaluation of genetic diversity of two leading blood stage malaria vaccine candidates of *P*. *vivax*, *Pvama1* complete ectodomain and *Pvmsp1*_*19*_ kDa region in three geographically diverse malaria-endemic regions of India.

In *Pvama1* high genetic diversity was observed in all the three regions of India, with substantial geographic variability and lower diversity in Chennai as compared to Rourkela and Nadiad. In Chennai *P*. *vivax* is a dominant species responsible for high burden of asymptomatic and submicroscopic malaria cases [6]. The prevalence of malaria in Chennai is lower as compared to Nadiad and Rourkela [6] which possibly contributed to the lower genetic diversity observed in Chennai. Genetic diversity in *Pvama1* from India, Iran, and Thailand was high compared to Korea and Venezuela. Lower diversity in Korea and Venezuela could be due to lower malaria transmission in these areas [8, 40, 41]. As polymorphism in *Pvama1* can help to reveal the *P*. *vivax* population history [42], higher genetic diversity observed in *Pvama1* from Asian countries supports the Southeast Asian origin of human *P*. *vivax* [43]. In *Pvama1* majority of diversity was detected in domain I (DI) in Rourkela and Nadiad as previously reported for both *P*. *vivax* and *P*. *falciparum* from other regions [44, 45], suggesting that DI is a dominant target of host immune responses [45–47]. Results from Tajima’s D, Fu and Li (D and F), and the MK test from Nadiad and Rourkela suggested that DI is under balancing selection, results in present study are consistent with previous studies that showed strong evidence of balancing selection in domain I [10, 36, 48, 49]. In Chennai negative Tajima’s D value were obtained which indicates population expansion or recent bottlenecks, were not significant here. No evidence of balancing selection was found in DII and DIII in *P*. *vivax* from Chennai, Nadiad and Rourkela as analyzed by Tajima’s D and Fu and Li (D and F) test which is in agreement with other studies from Venezuela, China-Myanmar Border, and PNG [10, 36, 45, 49]. In contrast, *P*. *vivax* population from Sri Lanka showed evidence of positive selection in domain II, reported as highly immunogenic [50, 51]. These results imply that the evolutionary pressure on *Pvama1* is different in geographically diverse regions of the world.

Polymorphic sites were distributed throughout *Pv*AMA1 ectodomain. However, domain I was the most variable, suggesting that this portion of the protein is potentially more exposed and accessible to host immune responses [36], which could have a significant effect on the immunological properties of the antigen. A total of 18 amino acid changes were observed in domain I, including codon changes at positions 172 (A/T) and 218 (L/V) which was not reported previously from India [25, 27]. At position 120, only two amino acid changes R/K were found in present study, whereas three amino acids R/K/S at position 120 were reported previously from India [25, 27]. A total of nine amino acid changes were found in domain II, six changes found in present study at position 253, 288, 352, 380, 384, 385 were not reported from Indian isolates previously and three changes at position 277, 368, 382 were reported from Rajasthan, northern part of India [27]. In domain III amino acid change at position 400 (K/R) found in Nadiad and Rourkela only, whereas amino acid change at position 438(R/H) was found in Chennai, Nadiad and Rourkela and reported previously in *P*. *vivax* isolates from Rajasthan [27]. In *Pv*AMA1 ectodomain 23 residues were located on one side, suggesting that this side might be targeted by host immune responses. As reported previously [36, 49], residue E145A, P210S, G253E, K352E and R438H mapped to predicted B-cell epitopes. In addition, residue E145A, important for immune evasion [49] was found in *P*. *vivax* isolates at all three study sites in India. In DII of PvAMA1 highly antigenic region (aa 290–307 SASDQPTQYEEEMTDYQK) during natural infection [52] was found to be conserved in Indian isolates. Furthermore, immunological studies indicate that DII of AMA1 is more immunogenic than DI during natural human infections and this region could be used as part of a sub-unit vaccine to prevent *P*. *vivax* malaria [12]. An effective malaria vaccine should include alleles (haplotypes) that can induce host immune responses against polymorphic antigens to cover the antigenic diversity [24, 53]. In India 55 *Pvama1* haplotypes were observed in present study and only few haplotypes were shared between the three Indian subpopulations. In 716 global dataset, 352 haplotypes were identified with no matches to the Sal1 reference strain, which has been used for immune-epidemiological studies and vaccine development. Malaria vaccine based on only reference strains may result in poor clinical efficacy due to high genetic diversity and strain specific immunity in vaccine candidates [54], which further shows the necessity to include alleles that are representative of natural parasite population. *F*_ST_ estimation between India and the closest geographical site Iran showed little genetic differentiation, which may be due to human population movement between the two countries. Only few haplotypes were shared between India, Sri Lanka and Iran indicating that similar allelic forms of *Pvama1* circulating in these regions. The complex network of *Pvama1* connected by rare admixed haplotypes and evidence of geographically restricted haplotypes suggest that covering *Pvama1* diversity would be more challenging [36]. Genetic diversity in *Pvama1* gene may contribute to antibody binding and escape, which needs further investigation to understand weather *Pvama1* haplotypes are antigenically distinct as previously reported for *Pf*AMA1 [55]. Genetic diversity may vary greatly in different malaria endemic regions, thus three study sites included in present study and limited number of positive samples could be a limiting factor.

*Pv*MSP1 is a merozoite surface antigen, The 42 kDa region breaks into two fragments 33 kDa and 19 kDa. The predicted structure shows that 33 kDa fragment covers the 19 kDa fragment. This observation could explain the diversity and balancing selection present in the 33 kDa fragment but not in 19 kDa fragment [13]. Sequence analysis of the *Pvmsp1*_*19*_ kDa fragment demonstrated that this region is highly conserved in *P*. *vivax* isolates from Chennai, Nadiad and Rourkela. A study conducted at same study sites reported high antibody response against *Pv*MSP1_19_ antigen as compared to *Pv*AMA1 in people living in these areas [28]. The *Pvmsp1*_*19*_ region was found to be conserved in many parts of India i.e. New Delhi (Delhi), Aligarh (Uttar Pradesh), Panjim (Goa), Panna (Madhya Pradesh) Sonapur (Assam), and Car Nicobar (Andaman & Nicobar Islands) [26]. Similar conserved amino acids in the *Pvmsp1*_*19*_ region were observed among India, China, Vanuatu and Mexico and found to be identical at the nucleotide level to the Belem and Salvador-1 types. Low genetic diversity and geographic clustering was observed in *Pvmsp1*_*19*,_ among the parasite populations from Turkey, Thailand, Bangladesh, South Korea, Sri Lanka, Singapore, Cambodia, and Myanmar suggesting that immune selection maintains similar *Pvmsp1*_*19*_ alleles around the globe.

A total of 14 *Pvmsp1*_*19*_ haplotypes was identified in 682 global isolates. Since negative Tajima’s D was observed in multiple populations, and only Turkey and Cambodia showed positive Tajima’s D values, which indicates that *Pvmsp1*_*19*_ region has undergone a recent bottleneck and lacks evidence of balancing selection. The level of selection pressure appears to vary on different parts of the protein in different *Plasmodium* species. In *P*. *falciparum*, *msp1*_*19*_ is under positive selection pressure and *Pvmsp1*_*33*_ is under purifying selection [56]. As reported previously [57] *Pvmsp1*_*19*_ region maintains low genetic diversity in geographically diverse malaria endemic regions shows that *Pvmsp1*_*19*_ based vaccines may work effectively worldwide. Multiple-allele immunogens appear to be protective against heterologous infection and vaccine constructs based on two or more representative strains and antigens may offer an effective solution to the problem of polymorphism [47, 58]. A recent study [58] indicates that recombinant protein containing *Pv*MSP1_19_ is advantageous, because of the improved cellular immune response.

In summary, this study presents the genetic diversity in *Pv*MSP1_19_ and *Pv*AMA1 vaccine candidate antigens in three geographically diverse malaria endemic regions of India and global dataset available in public domain. This is the first extensive study from India which contributes towards understanding the genetic diversity of *Pv*AMA*1* and *Pv*MSP1_19_ vaccine candidates. Highly conserved *Pvmsp1*_*19*_ region is observed in present study and previously reported high antibody response against *Pv*MSP1_19_ antigen in malaria endemic populations of India confirm it as one of the most promising malaria vaccine candidates. In contrast, exceptionally high genetic diversity is observed in *Pv*AMA*1* among Indian and global population. Here, domain I of *Pv*AMA1 is observed to be a dominant target of balancing selection. Overall, it seems that both the studied malaria antigens are under distinct selective pressure by the human immune system and thus genetic diversity in vaccine candidate antigens must be considered while developing a broadly efficacious malaria vaccine.

## Supporting information

S1 Fig Geographical map of India (map drawn by Author) showing three study sites. (PPTX)

S2 Fig Median-joining network of *Pvama1* between three geographically diverse populations of India. *Pvama1* ectodomain sequences from three geographical diverse malaria-endemic regions of India were used to create a median-joining network. This network represents the mutational paths connecting *Pvama1* haplotypes that may explain the observed sequence diversity. Each node represents one haplotype, node size indicates haplotype frequency and node color corresponds to the country of origin. Line length is proportional to genetic distance.
(PPTX)

S3 Fig Phylogenetic analysis of Indian *Pvama1* sequences. Neighbor-joining tree constructed using 100 *Pvama1* ectodomain sequences from India. Chennai sequences are in yellow, Nadiad sequences are in red and Rourkela sequences are in blue. The *Sal-1* reference sequence was also included and indicated as a triangle. The unrooted neighbor-joining tree was constructed using 10,000 bootstrap replicates and the branch lengths are in the same units as evolutionary distance used to infer the tree.
(PPTX)

S1 Table Oligonucleotides used for amplification and sequencing of *Pvama1* and *Pvmsp1*_*19*_. (PPTX)

S2 Table Pv*msp-1*_*19*_ and Pv*ama1* sequences included in this study. (PPTX)
