# Supplementary material for: Genetic diversity in two leading Plasmodium vivax malaria vaccine candidates AMA1 and MSP119 at three sites in India
Source: PLoS Negl Trop Dis. 2021 Aug 9;15(8):e0009652. doi: 10.1371/journal.pntd.0009652 (PMC8376102; doi:10.1371/journal.pntd.0009652)
Supplement: S1 Fig — (PPTX) [file pntd.0009652.s001.pptx]

## Slide 1
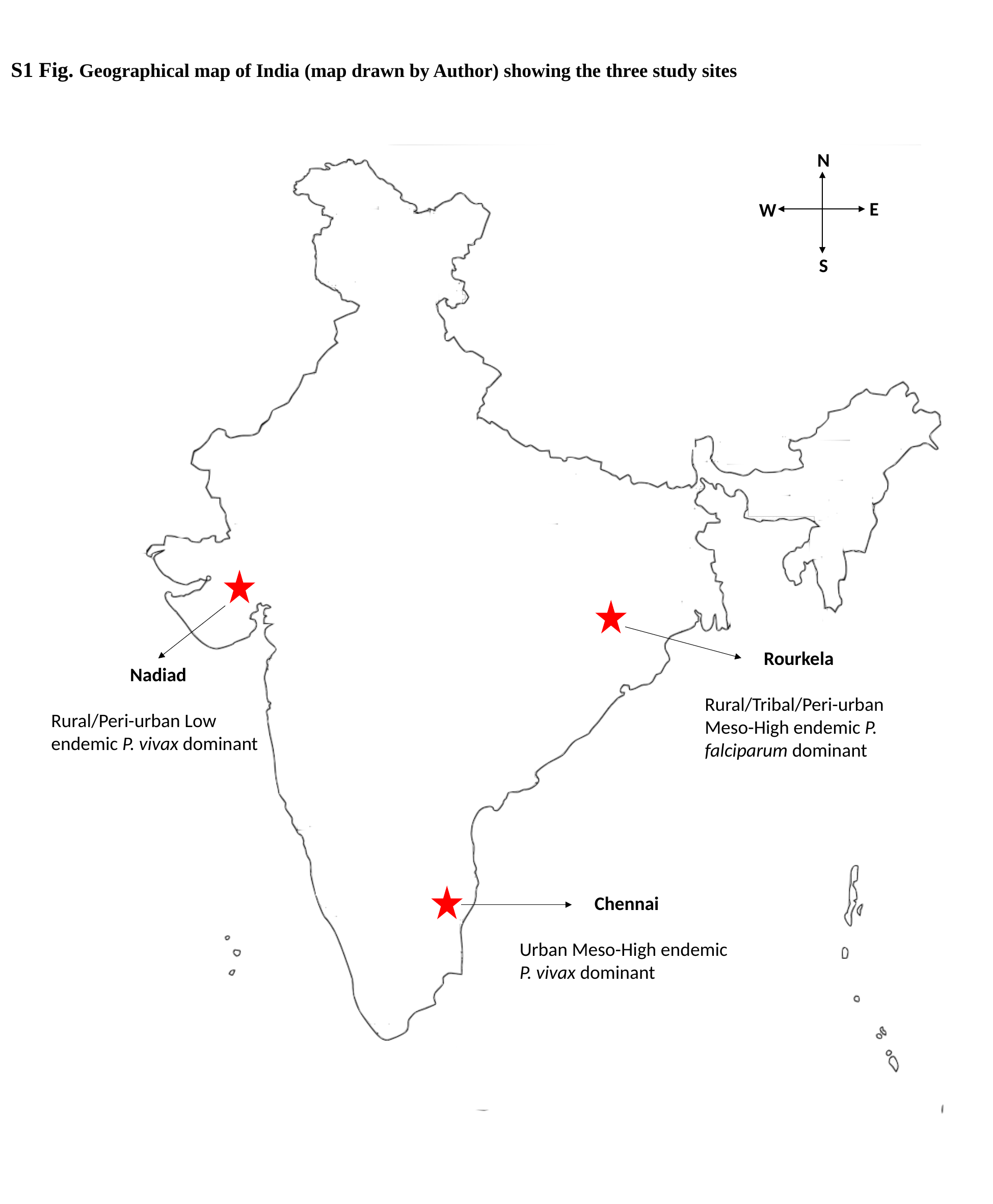

S1 Fig. Geographical map of India (map drawn by Author) showing the three study sites
N
E
W
S
Rourkela
Rural/Tribal/Peri-urban Meso-High endemic P. falciparum dominant
Nadiad
Rural/Peri-urban Low endemic P. vivax dominant
Chennai
Urban Meso-High endemic P. vivax dominant
