# Supplementary material for: Genetic diversity in two leading Plasmodium vivax malaria vaccine candidates AMA1 and MSP119 at three sites in India
Source: PLoS Negl Trop Dis. 2021 Aug 9;15(8):e0009652. doi: 10.1371/journal.pntd.0009652 (PMC8376102; doi:10.1371/journal.pntd.0009652)
Supplement: S2 Fig — Pvama1 ectodomain sequences from three geographical diverse malaria-endemic regions of India were used to create a median-joining network. This network represents the mutational paths connecting Pvama1 haplotypes that may explain the observed sequence diversity. Each node represents one haplotype, node size indicates haplotype frequency and node color corresponds to the country of origin. Line length is proportional to genetic distance. (PPTX) [file pntd.0009652.s002.pptx]

## Slide 1
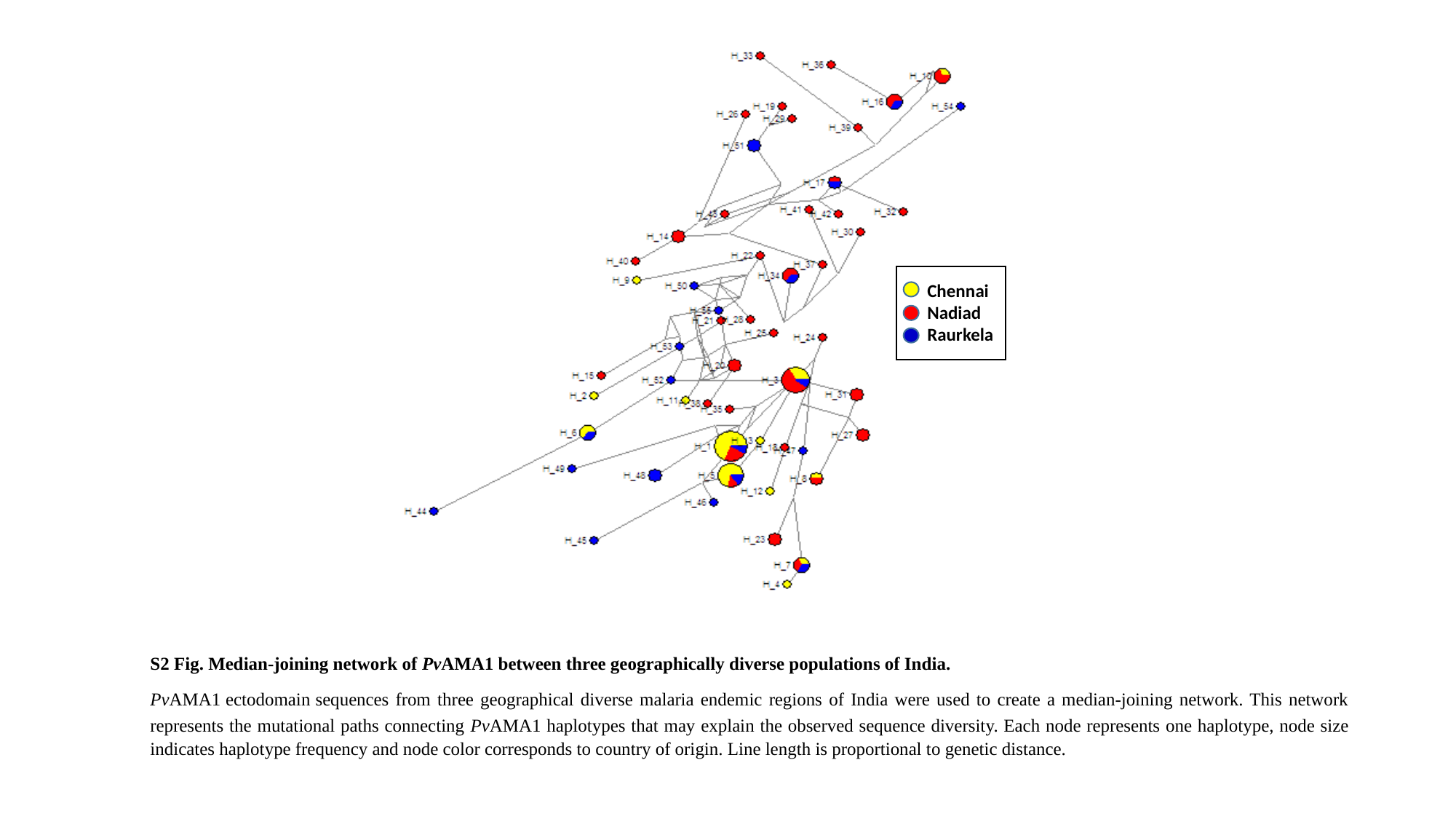

Chennai
Nadiad
Raurkela
S2 Fig. Median-joining network of PvAMA1 between three geographically diverse populations of India.
PvAMA1 ectodomain sequences from three geographical diverse malaria endemic regions of India were used to create a median-joining network. This network represents the mutational paths connecting PvAMA1 haplotypes that may explain the observed sequence diversity. Each node represents one haplotype, node size indicates haplotype frequency and node color corresponds to country of origin. Line length is proportional to genetic distance.
