# Supplementary material for: Genetic diversity in two leading Plasmodium vivax malaria vaccine candidates AMA1 and MSP119 at three sites in India
Source: PLoS Negl Trop Dis. 2021 Aug 9;15(8):e0009652. doi: 10.1371/journal.pntd.0009652 (PMC8376102; doi:10.1371/journal.pntd.0009652)
Supplement: S3 Fig — Neighbor-joining tree constructed using 100 Pvama1 ectodomain sequences from India. Chennai sequences are in yellow, Nadiad sequences are in red and Rourkela sequences are in blue. The Sal-1 reference sequence was also included and indicated as a triangle. The unrooted neighbor-joining tree was constructed using 10,000 bootstrap replicates and the branch lengths are in the same units as evolutionary distance used to infer the tree. (PPTX) [file pntd.0009652.s003.pptx]

## Slide 1
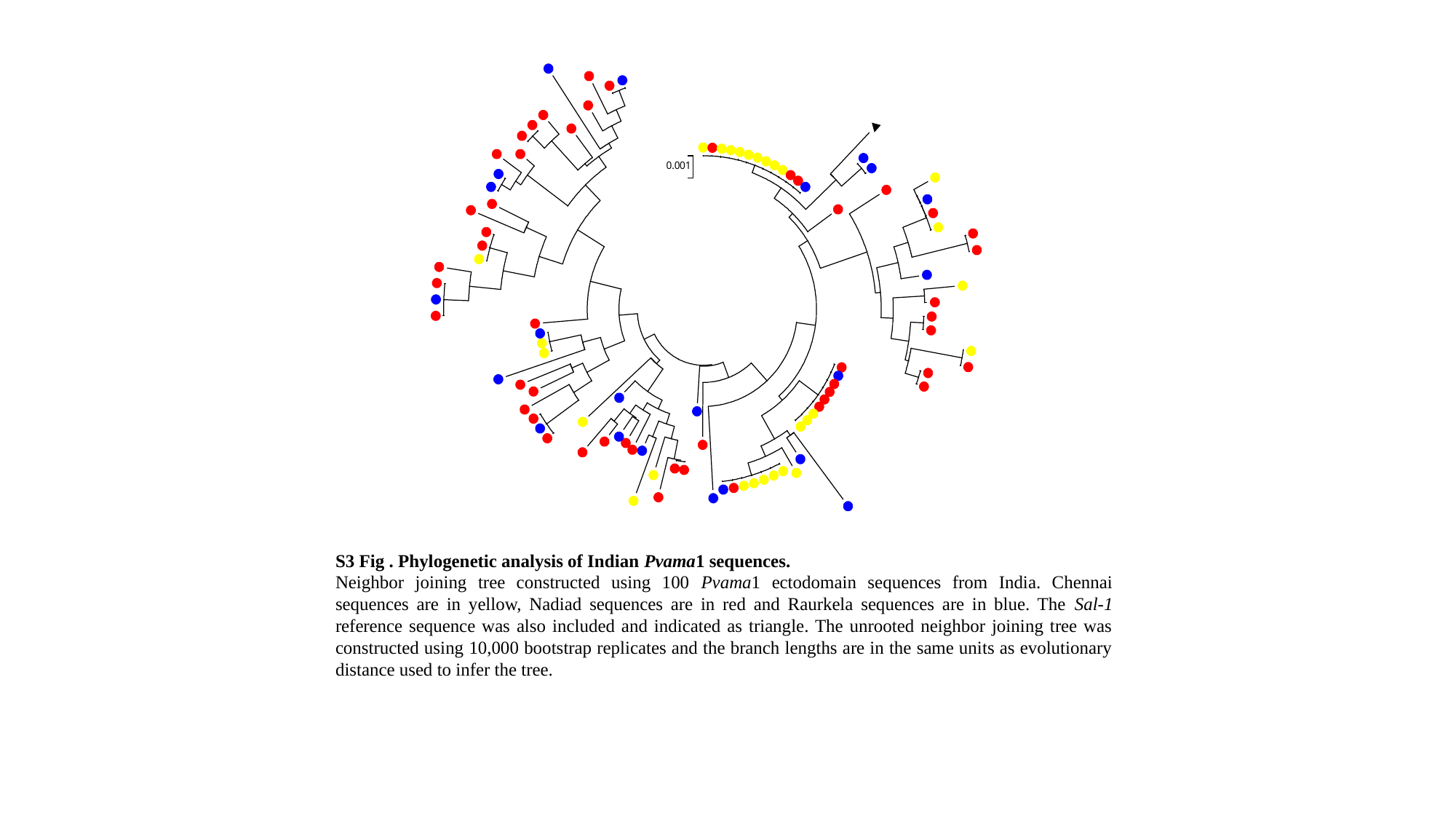

S3 Fig . Phylogenetic analysis of Indian Pvama1 sequences.
Neighbor joining tree constructed using 100 Pvama1 ectodomain sequences from India. Chennai sequences are in yellow, Nadiad sequences are in red and Raurkela sequences are in blue. The Sal-1 reference sequence was also included and indicated as triangle. The unrooted neighbor joining tree was constructed using 10,000 bootstrap replicates and the branch lengths are in the same units as evolutionary distance used to infer the tree.
