# Supplementary material for: Genetic diversity in two leading Plasmodium vivax malaria vaccine candidates AMA1 and MSP119 at three sites in India
Source: PLoS Negl Trop Dis. 2021 Aug 9;15(8):e0009652. doi: 10.1371/journal.pntd.0009652 (PMC8376102; doi:10.1371/journal.pntd.0009652)
Supplement: S1 Table — (PPTX) [file pntd.0009652.s004.pptx]

## Slide 1
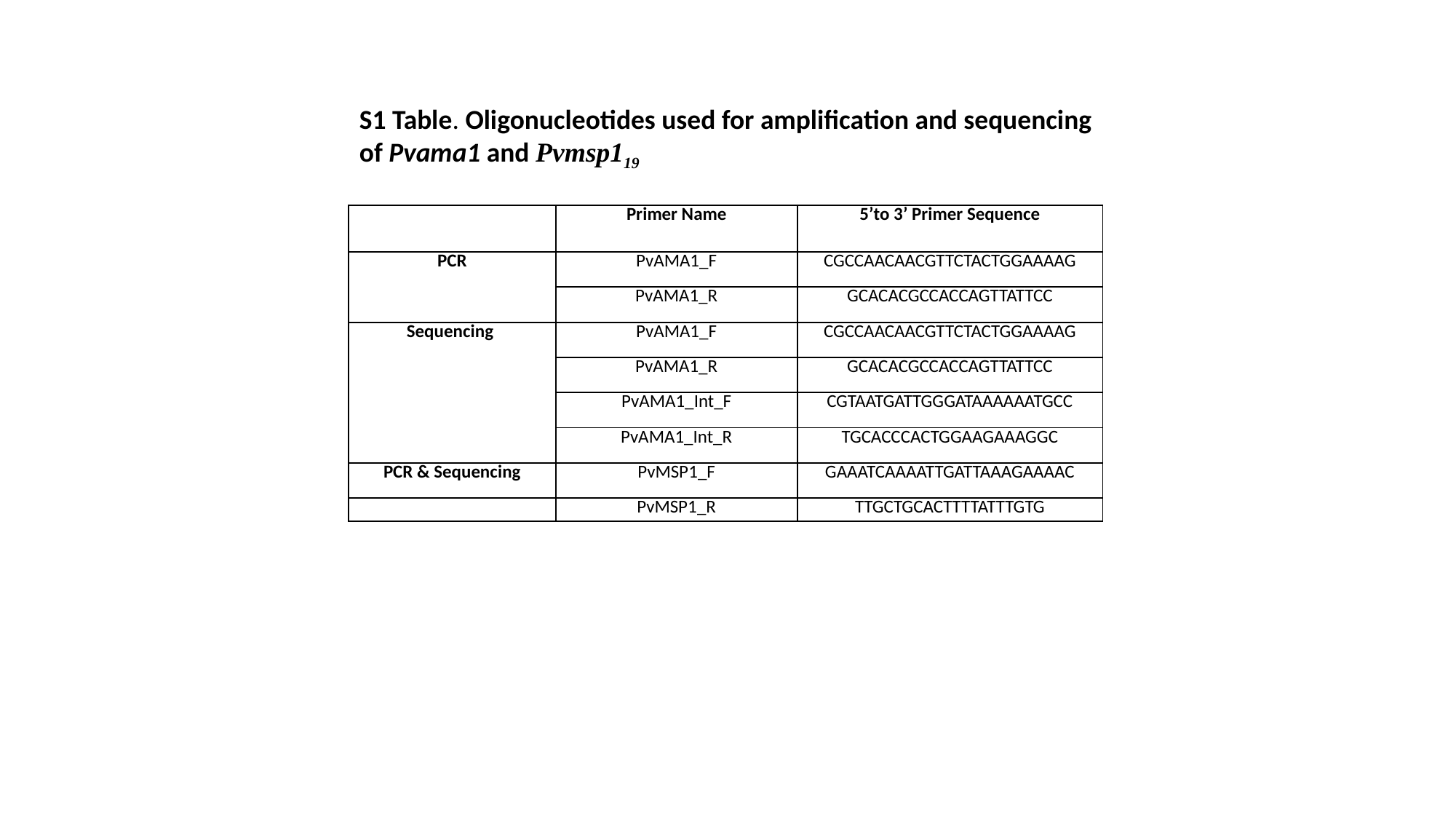

S1 Table. Oligonucleotides used for amplification and sequencing of Pvama1 and Pvmsp119
| | Primer Name | 5’to 3’ Primer Sequence |
| --- | --- | --- |
| PCR | PvAMA1\_F | CGCCAACAACGTTCTACTGGAAAAG |
| | PvAMA1\_R | GCACACGCCACCAGTTATTCC |
| Sequencing | PvAMA1\_F | CGCCAACAACGTTCTACTGGAAAAG |
| | PvAMA1\_R | GCACACGCCACCAGTTATTCC |
| | PvAMA1\_Int\_F | CGTAATGATTGGGATAAAAAATGCC |
| | PvAMA1\_Int\_R | TGCACCCACTGGAAGAAAGGC |
| PCR & Sequencing | PvMSP1\_F | GAAATCAAAATTGATTAAAGAAAAC |
| | PvMSP1\_R | TTGCTGCACTTTTATTTGTG |
