# Supplementary material for: Genetic diversity in two leading Plasmodium vivax malaria vaccine candidates AMA1 and MSP119 at three sites in India
Source: PLoS Negl Trop Dis. 2021 Aug 9;15(8):e0009652. doi: 10.1371/journal.pntd.0009652 (PMC8376102; doi:10.1371/journal.pntd.0009652)
Supplement: S2 Table — (PPTX) [file pntd.0009652.s005.pptx]

## Slide 1
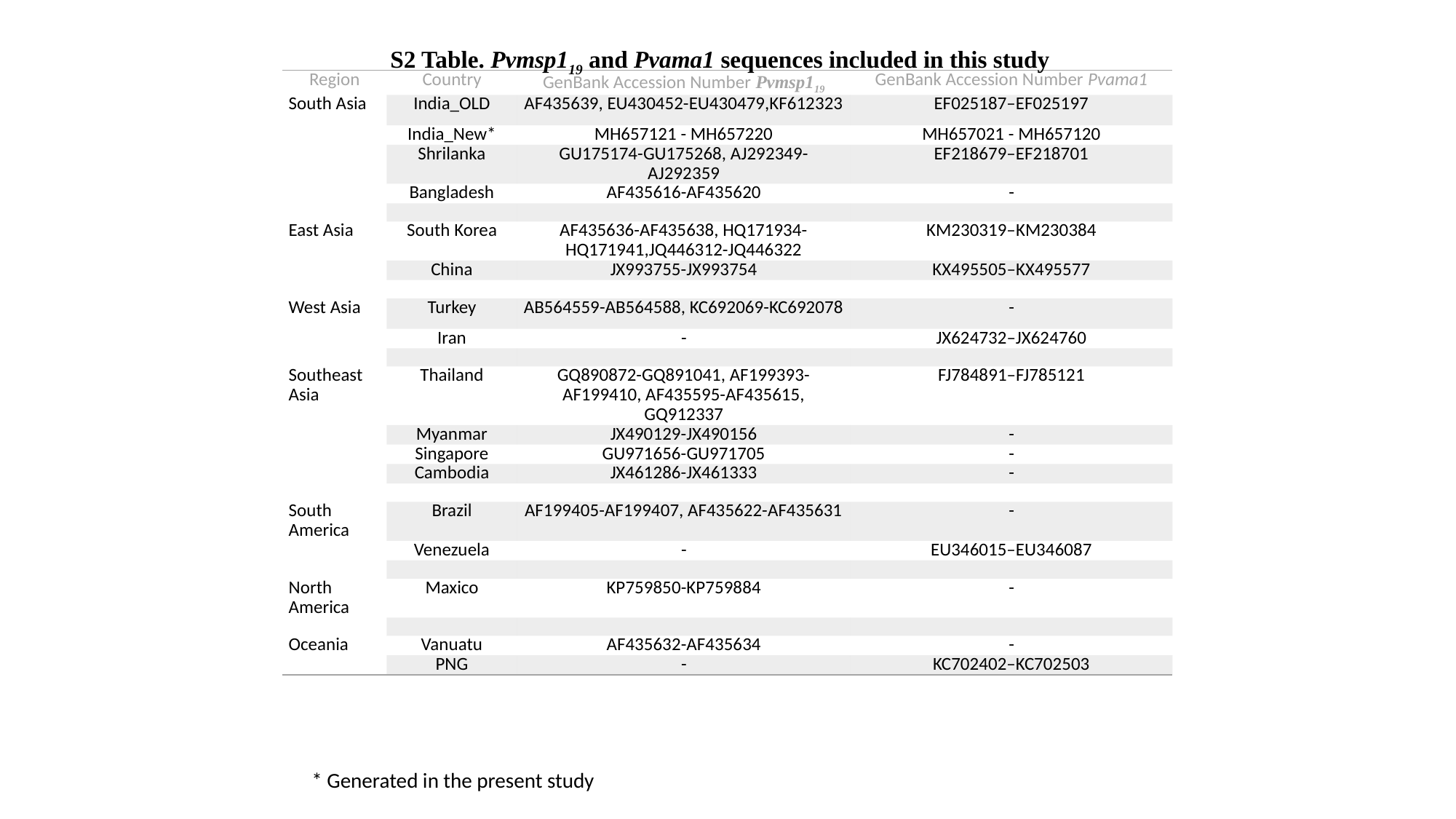

S2 Table. Pvmsp119 and Pvama1 sequences included in this study
| Region | Country | GenBank Accession Number Pvmsp119 | GenBank Accession Number Pvama1 |
| --- | --- | --- | --- |
| South Asia | India\_OLD | AF435639, EU430452-EU430479,KF612323 | EF025187–EF025197 |
| | India\_New\* | MH657121 - MH657220 | MH657021 - MH657120 |
| | Shrilanka | GU175174-GU175268, AJ292349-AJ292359 | EF218679–EF218701 |
| | Bangladesh | AF435616-AF435620 | - |
| | | | |
| East Asia | South Korea | AF435636-AF435638, HQ171934-HQ171941,JQ446312-JQ446322 | KM230319–KM230384 |
| | China | JX993755-JX993754 | KX495505–KX495577 |
| | | | |
| West Asia | Turkey | AB564559-AB564588, KC692069-KC692078 | - |
| | Iran | - | JX624732–JX624760 |
| | | | |
| Southeast Asia | Thailand | GQ890872-GQ891041, AF199393-AF199410, AF435595-AF435615, GQ912337 | FJ784891–FJ785121 |
| | Myanmar | JX490129-JX490156 | - |
| | Singapore | GU971656-GU971705 | - |
| | Cambodia | JX461286-JX461333 | - |
| | | | |
| South America | Brazil | AF199405-AF199407, AF435622-AF435631 | - |
| | Venezuela | - | EU346015–EU346087 |
| | | | |
| North America | Maxico | KP759850-KP759884 | - |
| | | | |
| Oceania | Vanuatu | AF435632-AF435634 | - |
| | PNG | - | KC702402–KC702503 |
* Generated in the present study
